# Therapeutic potential of *Codonopsis lanceolata* peel extract in premenstrual syndrome: insights into hormonal, immune, and microbial interactions

**DOI:** 10.1007/s10068-025-02003-w

**Published:** 2025-10-17

**Authors:** Hyeonjun Gwon, Hyeon Ji Kim, Ji-Woong Jeong, Daehyeop Lee, Joo Yun Kim, Jae Jung Shim, Jae-Hwan Lee

**Affiliations:** R & BD Center, Hy Co. Ltd., 22, Giheungdanji-ro 24beon-gil, Giheung-gu, 17086 Yongin-si Republic of Korea

**Keywords:** Premenstrual syndrome, Intestinal microbiota, prolactin, *Codonopsis lanceolata*

## Abstract

This study investigated the therapeutic potential of *Codonopsis lanceolata* peel extract (CPE) for premenstrual syndrome (PMS). The focus was on hormonal, immune, and microbial pathways. In vitro, CPE reduced nitric oxide production and suppressed IL-6 and TNF-α expression in LPS-stimulated macrophages. In pituitary GH3 cells stimulated with estradiol, CPE inhibited prolactin secretion. In a metoclopramide (MCP)-induced hyperprolactinemia mouse model, CPE decreased serum prolactin, PGE2, and inflammatory cytokines, while increasing PGE1 and FSH levels. Microbiome analysis showed that CPE enriched beneficial genera such as *Lactobacillus* and *Bifidobacterium* and reduced pro-inflammatory taxa including *Oscillibacter* and *Desulfovibrionaceae*. These microbial shifts were associated with improved inflammatory and hormonal markers. Overall, these findings suggest that CPE alleviates PMS symptoms by modulating endocrine function and regulating gut microbiota.

**Supplementary Information:**

The online version contains supplementary material available at 10.1007/s10068-025-02003-w.

## Introduction

Premenstrual syndrome (PMS), which affects a substantial proportion of women of reproductive age, manifests as recurrent physical and emotional symptoms that occur during the late luteal phase and subside with the onset of menstruation (Yonkers et al., 2008). The etiology involves complex interactions between fluctuating sex hormones and immune dysregulation. These include elevated prolactin levels, altered estrogen-to-progesterone ratios, and heightened inflammatory responses (Rapkin and Winer, 2009).

Systemic immune responses are important contributors to PMS pathophysiology. Pro-inflammatory cytokines such as IL-6 and TNF-α stimulate prostaglandin production, including PGE1, PGE2, and PGF2α. These mediators increase uterine contractility and activate pain pathways, which intensify cramps and discomfort (Hansen et al., 1999). Elevated prostaglandin levels may also disrupt neuroendocrine signaling and influence prolactin secretion (Bugajski, 1996). Prolactin, a pituitary hormone primarily associated with lactation, also regulates immune responses and emotional behavior. Dysregulation of prolactin has been observed in and may be caused by increased estrogen levels in the luteal phase (Bole-Feysot et al., 1998). High prolactin levels can disrupt the balance between estrogen and progesterone, contributing to emotional instability and heightened inflammatory sensitivity (Kappen et al., 2022).

Current treatments for PMS usually target hormones or inflammation. These include selective serotonin reuptake inhibitors, hormonal contraceptives, and Non-Steroidal Anti-Inflammatory Drug (NSAIDs). However, they are often linked to side effects and limited long-term efficacy. Plant-derived extracts with bioactive properties have attracted attention as alternatives for PMS treatment because they can affect both hormonal and immune pathways (Byeon et al., 2009; Chen et al., 2007). Polyphenols, in particular, can suppress prostaglandin synthesis, inhibit estrogen receptor activity, and reduce prolactin secretion in experimental models (Martinez and Moreno, 2000). *Codonopsis lanceolata* (*C. lanceolata*) has been traditionally used in East Asia, and its peel has been reported to contain bioactive compounds such as polyphenols and saponins (Hossen et al., 2016). However, its potential to improve PMS symptoms has not yet been investigated.

This study aimed to evaluate whether *C. lanceolata* peel extract (CPE) can alleviate PMS-related symptoms. To this end, a GH3 pituitary cell model stimulated with 17β-estradiol and a metoclopramide (MCP)-induced hyperprolactinemia mouse model were used, and the effects on prolactin, inflammatory cytokines, prostaglandins, and gut microbiota composition were assessed.

## Materials and methods

### Preparation of *Codonopsis lanceolata* peel extract

The dried *Codonopsis lanceolata* peels were obtained from Eosamae Deodeok Agricultural Cooperative (Hoengseong, Republic of Korea). The dried peels (100 g) were stirred for 18 h at 60  in 1 L of 50% ethanol (*w*/*v*). Extracted samples were filtered, and concentrated under reduced pressure using a rotary vacuum evaporator Eva‑05 (Daihan Scientific, Wonju, Korea) under reduced pressure. The concentrated extract was lyophilized, powdered, and stored at − 20℃ until use.

### Cell culture

Raw 264.7 murine macrophages and GH3 pituitary cells (ATCC, Manassas, VA, USA) were cultured under 37℃ and 5% CO_2_. Raw 264.7 cells were maintained in DMEM (Gibco, Waltham, MA, USA) with 10% fetal bovine serum (FBS) and 1% penicillin–streptomycin (P/S), while GH3 cells were maintained in F-12 K medium (Gibco) supplemented with 15% horse serum, 2.5% FBS, and 1% P/S.

### Measurement of nitric oxide (NO) and cytokines in Raw 264.7 cells

Raw 264.7 cells were seeded in 96-well plates (1 × 10^5^ cells/well) and stimulated with LPS (1 μg/mL) in the presence or absence of CPE (100 µg/mL) for 24 h. After incubation, 100 µL of culture supernatant was mixed with an equal volume of Griess reagent (1% sulfanilamide and 0.1% N-(1-naphthyl) ethylenediamine in 2.5% phosphoric acid) and incubated for 10 min at room temperature. Absorbance was measured at 540 nm using a BioTek® Synergy HT Microplate Reader (Santa Clara, CA, USA). Nitrite concentrations were calculated from a standard curve prepared with sodium nitrite (NaNO_2_).

In parallel, IL-6 and TNF-α in the same culture supernatants were quantified using mouse ELISA kits (BD OptEIA™, BD Biosciences, San Diego, CA, USA) according to the manufacturer’s instructions. Absorbance was measured at 450 nm, and cytokine concentrations were calculated from recombinant standard curves.

### Quantification of prolactin secreted from GH3 cells

GH3 cells were seeded into 12-well plates (2 × 10^5^ cells/well) and incubated for 24 h. The culture medium was then replaced with serum-free medium for starvation. Cells were pretreated with CPE at 10, 50, or 100 μg/mL for 2 h, followed by stimulation with 10 nM 17β-estradiol (E2; Sigma-Aldrich, St. Louis, MO, USA) for 24 h. The supernatants were collected, and prolactin levels were quantified using a Rat PRL ELISA Kit (LS-F3900, Lynnwood, WA, USA) according to the manufacturer’s instructions.

### Animal experiments

Fourteen-week-old female ICR mice (n = 36) were obtained from Central Lab Animal Inc. (Seoul, Republic of Korea) and housed under controlled environmental conditions (21–23℃, 45–65% humidity) with a 12 h light/dark cycle. After a one-week acclimatization period, the animals were randomly assigned to four groups (n = 9 per group): Control (CON), metoclopramide (MCP, 20 mg/kg/day), MCP + Prefemin (PFM, 100 mg/kg/day), and MCP + CPE (100 mg/kg/day). MCP was administered intraperitoneally every other day for 21 days to all groups except CON. PFM and CPE were dissolved in saline and given orally (200 μL/day) for 21 consecutive days, while the CON and MCP groups received the same volume of saline by oral gavage. Body weight and water intake were recorded weekly.

At the end of the study, blood, uterine, and cecum samples were collected. Blood was centrifuged at 3,000 × g for 20 min at 4 ℃ to obtain serum for biochemical analysis. Serum and cecum tissues were stored at − 80℃ until further analysis, and uterine samples were fixed in 10% neutral buffered formalin for histology. All animal procedures were approved by the Institutional Animal Care and Use Committee (IACUC) of hy Co., Ltd., Seoul, Republic of Korea (approval number: AEC-2024–0003-Y).

### Histological analysis

The fixed uterine tissues from mouse were embedded in paraffin wax and sectioned. Then, tissues sections were stained with hematoxylin & eosin (H&E) by DooYeol Biotech (Seoul, Republic of Korea). After H&E staining, the section images were taken by MoticDSAssistant (Motic VM V1 Viewer 2.0). Then, endometrial thickness of uterine was evaluated (ImageJ 2.1.0).

### Measurement of hormones and pro-inflammatory cytokines in serum

Serum levels of prolactin, follicle-stimulating hormone (FSH), and pro-inflammatory cytokines IL-1β, IL-6, and TNF-α were analyzed using the Luminex® Multiplex Assay system (Thermo Fisher Scientific, Carlsbad, CA) at LABISKOMA (Seoul, Republic of Korea). Prostaglandin E1 (PGE1) and Prostaglandin E2 (PGE2) were measured using ELISA Kits according to the manufacturer's instructions (LS-F28568 and LS-F32354, LS Bio, Lynnwood, WA, USA).

### Metagenomic analysis

Total DNA was isolated from the cecum and microbiota composition was confirmed by 16S rDNA sequencing using a next-generation sequencing platform (Illumina, San Diego, CA, United States). The universal primer pairs used for sequencing were: V3-F: 5′-TCGTCGGCAGCGTC AGATGTGTATAAGAGAC AGCCTACGGGNG GCWGCAG-3′ and V4-R: 5′-GTCTCGTGGGCTC GGAGATGTGTATAAGA GACAGGACTACHV GGGTATCTAATCC-3′. DNA extraction and 16S rDNA sequencing were performed at Macrogen (Seoul, South Korea).

Amplicon sequence data were processed using QIIME2 (version 2023.9). Raw reads were demultiplexed with the q2-demux plugin, and low-quality sequences were filtered and denoised using the DADA2 plugin to generate amplicon sequence variants (ASVs). MAFFT was used to align ASVs, and a rooted phylogenetic tree was constructed with FastTree 2 for downstream phylogenetic analyses. Taxonomic classification was performed using the q2-feature-classifier plugin with a pre-trained SILVA 138 reference database (99% identity threshold). Alpha diversity metrics including Faith’s Phylogenetic Diversity (Faith’s PD), and Observed Features were calculated to evaluate within-sample microbial diversity. Group differences in alpha diversity were tested using the non-parametric Kruskal–Wallis test. Beta diversity was assessed based on unweighted and weighted UniFrac distance matrices, and visualized through Principal Coordinates Analysis (PCoA). Differences in overall microbial community composition between groups were statistically evaluated using PERMANOVA (Permutational Multivariate Analysis of Variance). For taxonomic composition analysis, relative abundances of microbial taxa were compared across groups. Taxa present at ≥ 1% relative abundance in at least one group were defined as major taxa, while the remaining were grouped as minor taxa. Differentially abundant taxa were identified using LEfSe (Linear Discriminant Analysis Effect Size) with a significance threshold of LDA score > 3.0. Correlations between the relative abundance of gut microbiota and the biochemical indicators were calculated using Spearman’s rank correlation coefficient in the R software package (Version 3.6.6.). All NGS data were deposited in the NCBI Sequence Read Archive under accession code PRJNA1288695.

### Statistical analyses

The results were presented as mean ± standard deviation at the 95% confidence. Statistical differences among multiple groups were analyzed using one-way analysis of variance (ANOVA) followed by Tukey’s multiple comparison test using GraphPad Prism v5 (San Diego, CA, USA).

## Results and discussion

This study aimed to evaluate whether *C. lanceolata* peel extract (CPE) alleviates PMS-related symptoms by regulating prolactin secretion and inflammatory responses. Additionally, its effects on gut microbiota composition were examined to explore potential microbiome-mediated mechanisms.

**Fig. 1:**
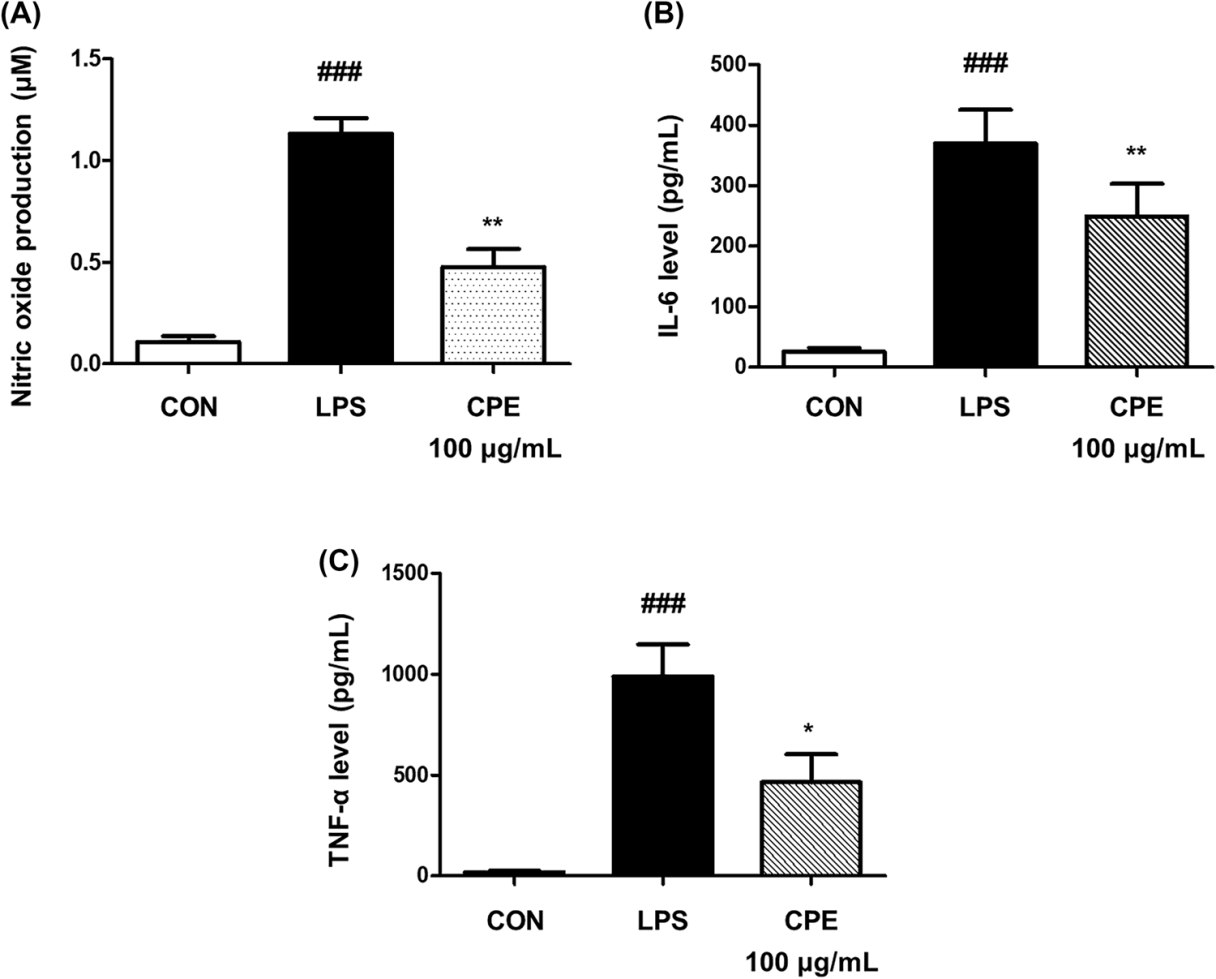
Anti-inflammatory effects of CPE on LPS-induced Raw 264.7 macrophages. **A** Nitric oxide (NO) production in Raw 264.7 cells stimulated with LPS (1 μg/mL) and treated with CPE. Levels of pro-inflammatory cytokines (**B**) IL-6 and (C) TNF-α following CPE treatment in LPS-stimulated cells. The results are expressed as the mean ± standard deviation. ^###^*p* < 0.001 compared with CON group. *p < 0.05, and **p < 0.01 compared with LPS group. *CON* Control group, *LPS* lipopolysaccharide-stimulated group, *CPE* LPS-stimulated group treated with *Codonopsis lanceolata* 50% ethanol extract

The anti-inflammatory potential of CPE was evaluated in LPS-stimulated Raw 264.7 macrophages. CPE treatment significantly reduced nitric oxide (NO) production, from 1.13 ± 0.13 µM in the LPS group to 0.48 ± 0.15 µM with CPE (*p* < 0.01; Fig. 1A), corresponding to a 57.7% decrease. CPE also suppressed the secretion of pro-inflammatory cytokines. IL-6 levels declined from 369.82 ± 55.63 pg/mL (LPS) to 249.90 ± 53.14 pg/mL (*p* < 0.01), representing a 32.4% reduction, while TNF-α levels decreased from 989.72 ± 158.00 pg/mL to 464.46 ± 138.57 pg/mL (*p* < 0.05), corresponding to a 53.1% reduction (Fig. 1B, C). These results demonstrate that CPE inhibits the production of inflammatory mediators, including nitric oxide and pro-inflammatory cytokines, in activated macrophages. No cytotoxicity was observed in either Raw 264.7 or GH3 cells at the tested concentration, confirming that the inhibitory effects were not attributable to reduced cell viability (Figure S1). This is consistent with previous reports that *Codonopsis lanceolata* extracts rich in polyphenols such as luteolin, attenuate LPS-induced inflammation by blocking iNOS, COX-2, and NF-κB activation (Choi et al., 2023). Since chronic low-grade inflammation has been implicated in PMS symptoms such as breast pain, swelling, and headaches (Bertone-Johnson et al., 2014), the inhibition observed with CPE suggests potential benefits for these conditions. However, the present study alone cannot confirm such effects, and further investigations, including clinical studies, are required.

**Fig. 2:**
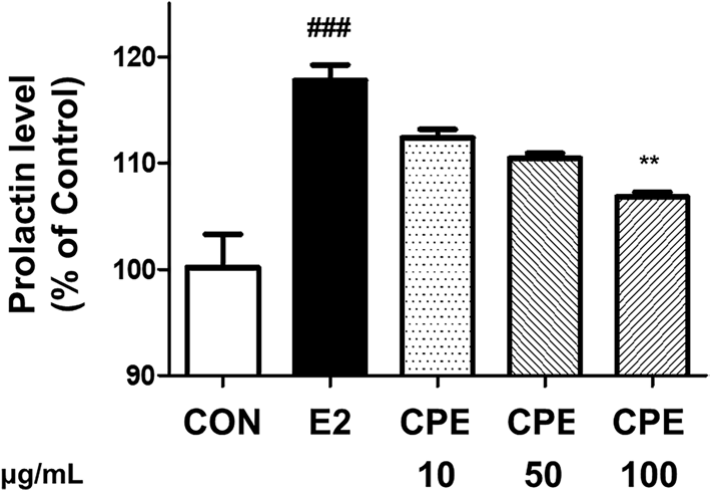
Dose-dependent effects of CPE on prolactin production in GH3 pituitary cells. Data are presented as mean ± standard deviation. ^###^*p* < 0.001 compared with CON group. **p < 0.01 compared with E2 group. *CON* control, *E2* 10 nM 17β-estradiol-stimulated group, *CPE* E2-stimulated group treated with *Codonopsis lanceolata* 50% ethanol extract

Another hallmark of PMS is hormonal imbalance, often involving elevated prolactin. This elevation has been linked to symptoms such as breast tenderness and mood changes. To examine whether CPE can modulate hormone secretion, its effect on prolactin release was evaluated in GH3 rat pituitary cells. As expected, stimulation with 17β-estradiol (E2) markedly increased prolactin secretion, reflecting estrogen’s known role in upregulating prolactin during the luteal phase (Frasor and Gibori, 2003). CPE treatment dose-dependently blunted this estrogen-induced prolactin release (Fig. 2). High-dose CPE significantly returned prolactin levels despite the presence of E2, indicating that CPE can counteract estrogen’s prolactin-elevating effect. This result suggests a potential mechanism by which CPE might help restore hormonal balance relevant to PMS. By keeping prolactin in check, CPE could mitigate PMS symptoms associated with hyperprolactinemia. In this regard, CPE shows a similar functional outcome to the known herbal remedy *Vitex agnus-castus* (Prefemin, PFM), which is widely used to treat PMS by suppressing excessive prolactin secretion (Puglia et al., 2023). Notably, PFM acts via dopaminergic mechanisms on the pituitary to inhibit prolactin release. Although the exact mode of action of CPE is yet to be elucidated, its effectiveness in the GH3 cell assay hints that it may influence pituitary lactotroph cells directly or modulate estrogen receptor signaling. By dampening estrogen-driven prolactin overproduction, CPE could help prevent the cascade of luteal phase defects associated with high prolactin, such as insufficient progesterone and associated mood disturbances (Seidlova-Wuttke and Wuttke, 2017). This endocrine-modulating capacity warranted further in vivo validation in a PMS model.

**Fig. 3:**
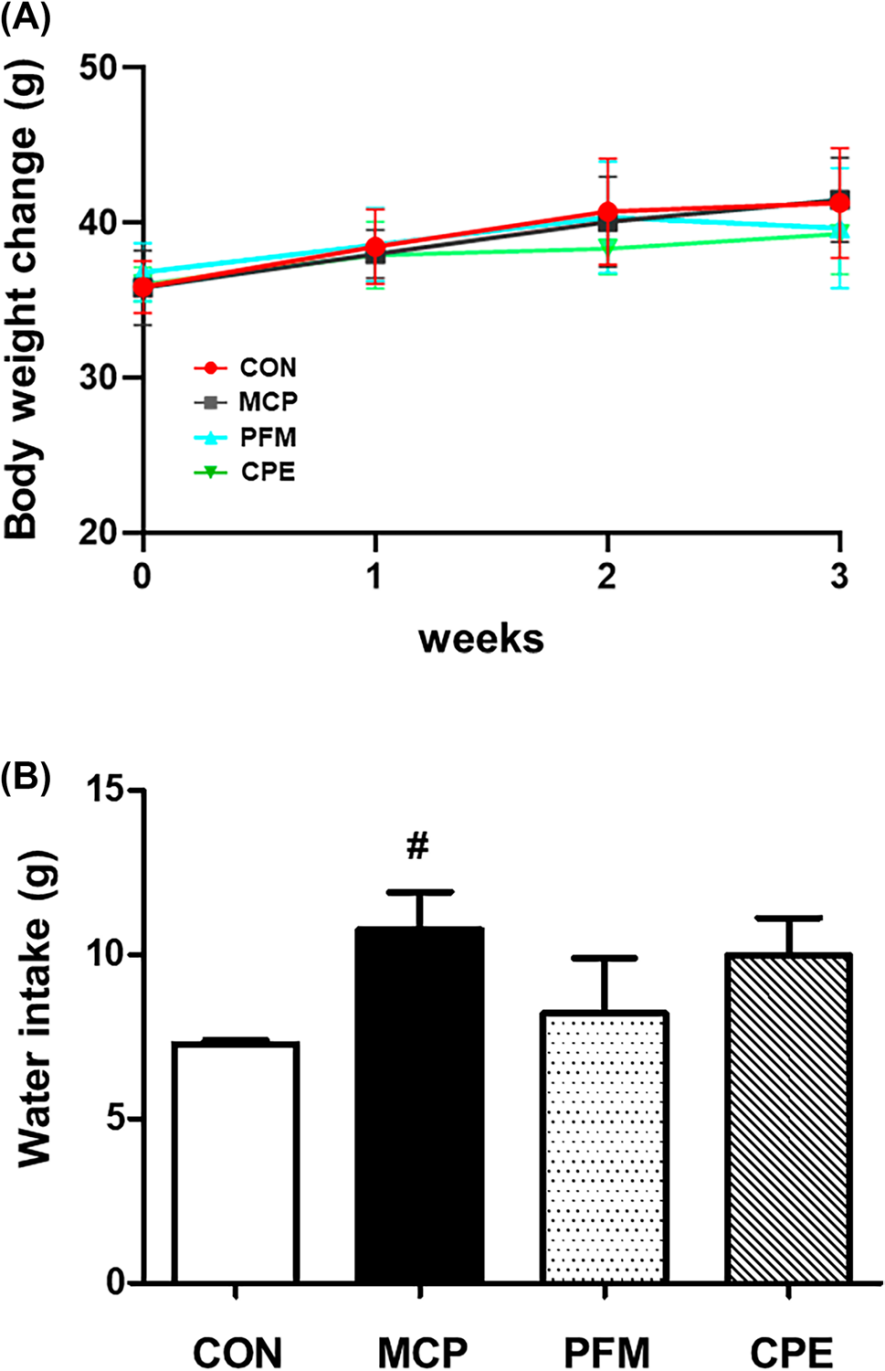
Changes in body weight and water intake in the MCP-induced hyperprolactinemia mouse model. **A** Body weight (g) of mice during the experimental period. **B** Water intake (g) among experimental groups. Data are presented as mean ± standard deviation. ^#^*p* < 0.05 compared with the CON group. *CON* control group, *MCP* metoclopramide-induced group, *PFM* MCP-induced group treated with prefemin, *CPE* MCP-induced group treated with *Codonopsis lanceolata* 50% ethanol extract

**Fig.4:**
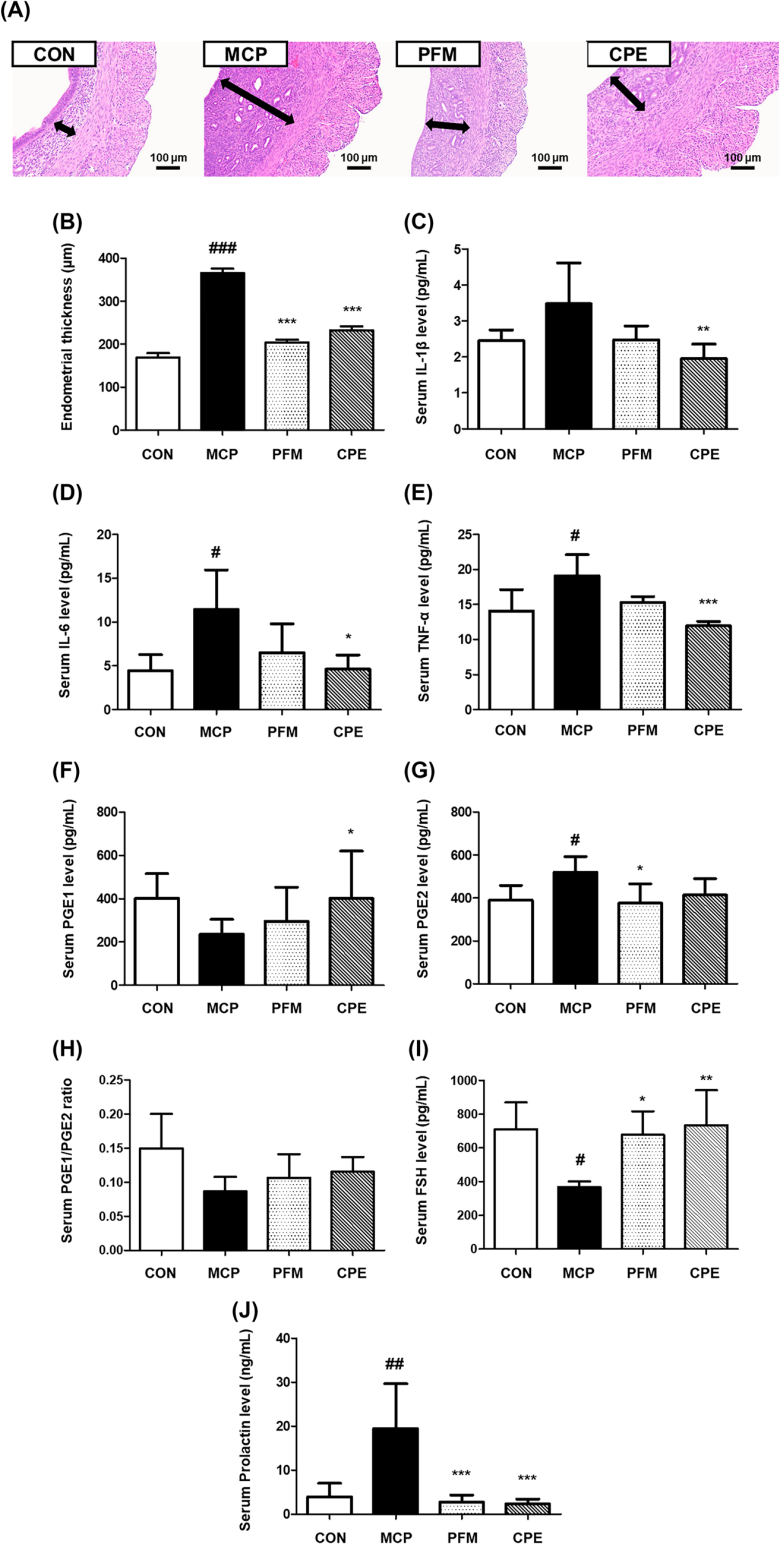
Histological and hormonal effects of PFM and CPE in MCP-induced PMS model mice. **A** H&E stained uterine tissues (× 200 magnification, arrows point to the endometrium). **B** Endometrial thickness of the uterus. Serum levels of inflammatory and hormonal markers including: **C** IL-1β, **D** IL-6, **E** TNF-α, **F** PGE1, **G** PGE2, **H** PGE1/PGE2 ratio, **I** FSH, and **J** prolactin. Data are represented as mean ± standard deviation. ^#^*p* < 0*.*05, ^##^*p* < 0*.*01 and ^###^*p* < 0*.*001 compared with CON group. *p < 0*.*05, **p < 0*.*01, and ***p < 0.001 compared with MCP group. *CON* control group, *MCP* metoclopramide-induced group, *PFM* MCP-induced group treated with prefemin, *CPE* MCP-induced group treated with *Codonopsis lanceolata* 50% ethanol extract

To translate the in vitro findings to a physiological context, a mouse model of hyperprolactinemia was employed using metoclopramide (MCP), a dopamine antagonist that elevates prolactin levels and mimics certain endocrine features of PMS, notably increased prolactin and resultant hormonal imbalances (Milewicz and Jedrzejuk, 2006). Although no animal model perfectly replicates human PMS, MCP-induced hyperprolactinemia induces analogous symptoms, including luteal phase disruptions and affective changes. Overall health indicators such as body weight were not significantly affected by any treatment over the study period (Fig. 3A), suggesting that neither MCP nor the tested supplements caused adverse effects on general growth. MCP did cause a modest increase in water intake compared to controls, consistent with polydipsia sometimes observed in hyperprolactinemic states (Kaufman et al., 1981). Interestingly, both PFM and CPE showed a tendency to normalize water consumption (Fig. 3B), although these changes were not statistically significant. A more pronounced physiological change was observed in the uterus: MCP treatment led to significant endometrial thickening (365.70 ± 25.04 µm vs. 169.36 ± 24.38 µm in control mice, *p* < 0.001). This hyperplasia is likely due to prolactin’s interaction with reproductive hormone balance, possibly prolonging the estrogen-driven proliferative effect on the endometrium. In the CPE group, endometrial thickness was reduced to 232.60 ± 22.34 µm (a 36.4% reduction from MCP, *p* < 0.001), while PFM reduced it to 203.89 ± 15.91 µm (*p* < 0.001 vs MCP) (Fig. 4A, B). These improvements indicate that both treatments mitigated the downstream estrogenic effects of hyperprolactinemia on uterine tissue. These findings align with the known ability of PFM to correct luteal phase defects and rebalance hormonal signals in hyperprolactinemic conditions (Haerifar et al., 2020). Comparable efficacy from CPE suggests that it may likewise modulate the hormonal environment to prevent abnormal endometrial proliferation, an important consideration since PMS patients often experience menstrual irregularities and endometrial sensitivity due to hormonal fluctuations.

A key feature of PMS is an imbalance in inflammatory mediators, including prostaglandins that drive pelvic cramping and systemic cytokines linked to mood symptoms. The MCP-induced hyperprolactinemia model established in this study was associated with a systemic pro-inflammatory state. MCP-induced hyperprolactinemia triggered a systemic pro-inflammatory state, with serum cytokines elevated compared to controls: IL-1β increased from 2.45 ± 0.30 to 3.48 ± 1.14 pg/mL (n.s), IL-6 from 4.44 ± 1.84 to 11.42 ± 4.52 pg/mL (*p* < 0.05), and TNF-α from 14.10 ± 3.03 to 19.08 ± 3.04 pg/mL (*p* < 0.05). Chronic hyperprolactinemia is known to promote cytokine release and create neuroimmune stress resembling PMS and PMDD (premenstrual dysphoric disorder) (Bertone-Johnson et al., 2014). Treatment with CPE significantly decreased all three cytokines toward normal levels: IL-1β decreased to 1.95 ± 0.40 pg/mL (43.9% reduction vs MCP, *p* < 0*.*01), IL-6 to 4.62 ± 1.63 pg/mL (59.6% reduction, *p* < 0*.*05), and TNF-α to 11.97 ± 0.62 pg/mL (37.3% reduction, *p* < 0*.*001) (Fig. 4C–E). These data corroborate the strong anti-inflammatory capacity of CPE observed in vitro, now demonstrated in vivo. PFM also showed an anti-inflammatory trend but with a narrower scope: PFM decreased IL-1β, IL-6, and TNF-α but that reduction did not reach statistical significance. This differential effect suggests that while both treatments can counteract certain inflammatory aspects of hyperprolactinemia, CPE may have a broader impact on the cytokine network, potentially due to its diverse bioactive constituents (polyphenols and polysaccharides) acting on multiple immune pathways (Vieira et al., 2024).

In tandem with cytokine normalization, CPE also corrected the imbalance in prostaglandin profiles caused by MCP. MCP-induced mice showed unstable prostaglandin ratio characteristic of inflammatory pain states, prostaglandin E1 (PGE1) was suppressed (215.07 ± 53.45 pg/mL vs 380.51 ± 113.26 pg/mL in controls) while prostaglandin E2 (PGE2) was elevated (520.20 ± 72.44 pg/mL vs 389.95 ± 67.45 pg/mL in controls) (*p* < 0.05). This shift reduced the PGE1/PGE2 ratio to 0.09 ± 0.02 in MCP-only mice, compared to 0.15 ± 0.05 in controls. PGE2 is a pro-inflammatory mediator that can exacerbate uterine cramps and mood symptoms, whereas PGE1 has vasodilatory and anti-inflammatory effects. Thus, a low PGE1/PGE2 ratio aggravates PMS symptoms. Treatment with CPE restored PGE1 levels (445.95 ± 211.28 pg/mL) and reduced PGE2 (414.49 ± 74.95 pg/mL), increasing the PGE1/PGE2 ratio to 0.12 ± 0.02 (Fig. 4F–H). Similarly, PFM adjusted the prostaglandin ratio to 0.11 ± 0.03. These shifts indicate that both CPE and PFM help rebalance prostaglandin production, which is significant because prostaglandins mediate many PMS symptoms such as cramps, breast tenderness, and headaches. By increasing the relative abundance of the less inflammatory PGE1 and curbing excess PGE2, CPE could alleviate PMS-related discomfort. Notably, these effects on prostaglandins are in line with CPE’s observed capacity to inhibit COX-2 and downstream PGE2 in the macrophage model (Choi et al., 2023), reinforcing that the extract’s bioactives reach target tissues in vivo to modulate inflammatory mediator synthesis.

Hyperprolactinemia is known to disrupt the reproductive hormonal axis (Serri et al., 2003). In MCP model, serum follicle-stimulating hormone (FSH) was significantly decreased in MCP-treated mice (368.23 ± 31.99 pg/mL) compared to healthy controls (710.28 ± 159.04 pg/mL) (*p* < 0.01), reflecting suppression of the hypothalamic-pituitary–gonadal (HPG) axis. In addition, elevated prolactin levels are known to inhibit GnRH release, leading to decreased FSH and LH secretion and a blunted luteal phase, thereby explaining the reduction of FSH levels observed in hyperprolactinemia-induced MCP models (Kaiser, 2012). Both CPE and PFM interventions successfully restored FSH levels in MCP-treated mice. CPE raised FSH to 735.68 ± 206.55 pg/mL (*p* < 0.01), and PFM to 677.72 ± 139.84 pg/mL (*p* < 0.05), not significantly different from control values (710.28 ± 159.04 pg/mL) (Fig. 4I). Normalization of FSH indicates reactivation of the HPG axis, likely due to lowered prolactin. MCP also induced marked hyperprolactinemia (Carroll and Steiner, 1978) (19.45 ± 10.21 ng/mL vs 3.96 ± 3.08 ng/mL in controls, *p* < 0.01). This elevated prolactin mirrors the condition seen in certain PMS patients and in pathological hyperprolactinemia, which can cause mood disturbances and reproductive dysfunction (Carroll and Steiner, 1978). Impressively, CPE supplementation reduced prolactin in MCP mice to 2.32 ± 1.12 ng/mL (*p* < 0.001), corresponding to an 88.1% reduction vs MCP. PFM had a comparable effect, lowering prolactin to 2.70 ± 1.64 ng/mL (86.1% reduction vs MCP, *p* < 0.001) (Fig. 4J). Both treatments brought prolactin back to the baseline range, consistent with the known prolactin-suppressing action of PFM in both animal models and clinical settings (van Die et al., 2013). The fact that CPE matched the efficacy of PFM in this regard is notable, suggesting CPE contains compounds that either enhance dopaminergic tone or otherwise act on pituitary lactotrophs to limit prolactin release. Taken together, the hormonal data show that CPE can reverse the endocrine perturbations of hyperprolactinemia by normalizing prolactin and secondarily normalizes FSH, indicating recovery of normal ovarian signaling. By restoring hormonal balance, CPE addresses a core aspect of PMS pathophysiology, as hormone fluctuations and imbalances underlie many PMS symptoms.

**Fig. 5:**
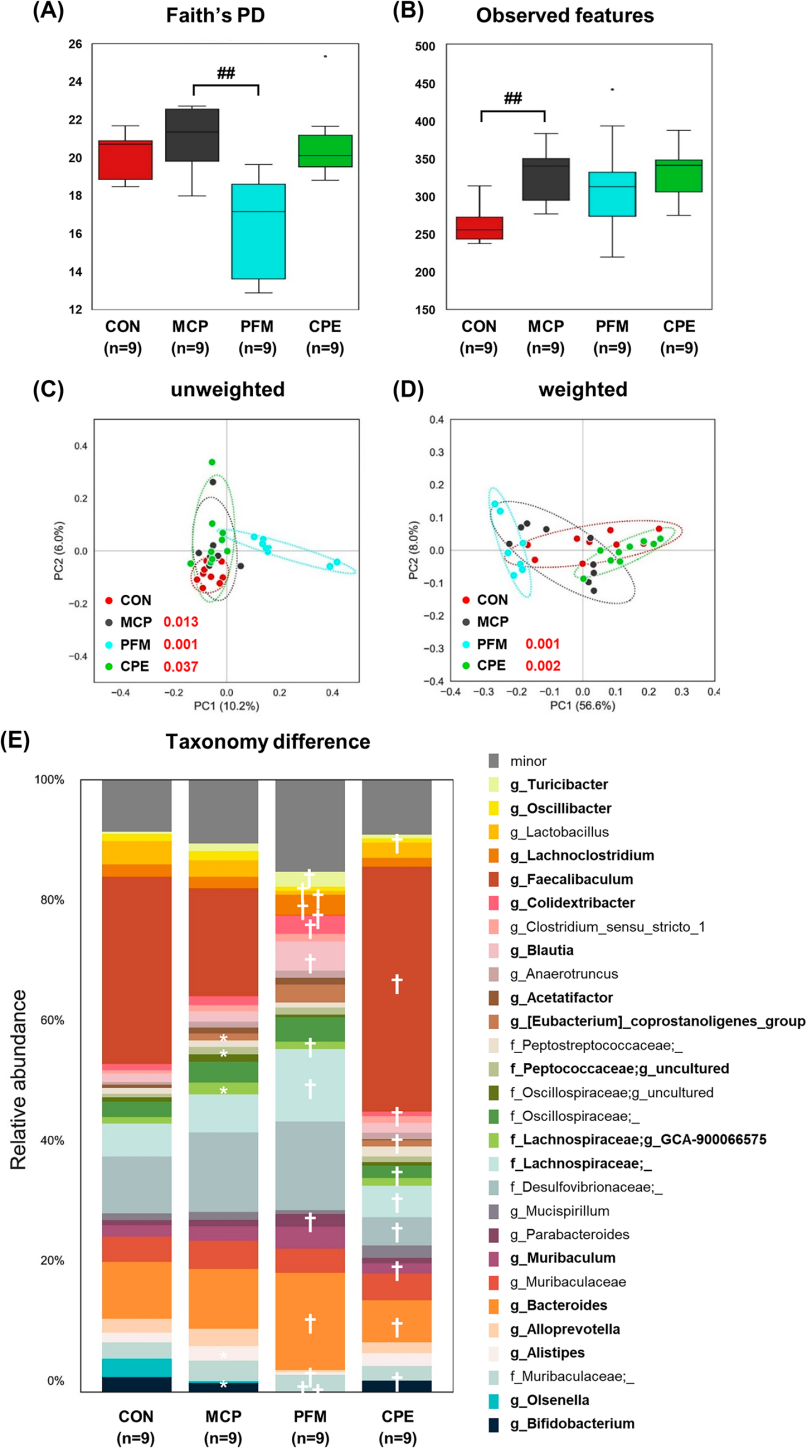
Diversity and taxonomic alterations in the intestinal microbiota of MCP-induced ICR mice and treatment groups. Alpha diversity measured by **A** Faith’s phylogenetic diversity (Faith’s PD) and **B** Observed features. ## *p* < 0*.*01 compared with MCP group. Beta diversity assessed using **C** unweighted and **D** weighted UniFrac distances. Statistical comparisons were performed using PERMANOVA between CON vs. MCP and MCP vs. PFM or CPE groups. **E** Taxonomic differences at the genus level, focusing on taxa with relative abundance > 1%. *LDA score > 3.0 vs. CON group; ^†^LDA score > 3.0 vs. MCP group. *CON* control group, *MCP* metoclopramide-induced group, *PFM* MCP-induced group treated with prefemin, *CPE* MCP-induced group treated with *Codonopsis lanceolata* 50% ethanol extract

Beyond immune and endocrine factors, recent research has highlighted the gut microbiome’s role in women’s health and even in mood regulation. The effect of CPE on gut microbiota composition in this PMS model was therefore examined as a novel angle to understand its multi-modal action. Gut microbiota changes were analyzed by 16S rRNA sequencing, which revealed that MCP-induced hyperprolactinemia altered both alpha and beta diversity. Alpha diversity (Faith’s phylogenetic diversity (PD) and Observed features) was increased in the MCP group (Fig. 5A, B). This indicates that MCP-induced hyperprolactinemia promoted dysbiosis, with an expansion of taxa linked to pro-inflammatory states rather than a healthy increase in commensal richness. Interestingly, PFM treatment tended to reduce alpha diversity relative to MCP (though not fully to control levels), suggesting a selective suppression of certain taxa. In contrast, CPE-treated mice maintained a more balanced diversity profile, preserving microbial richness while preventing overgrowth of harmful taxa. For beta diversity, unweighted UniFrac principal coordinates analysis showed clear clustering of microbial communities by treatment group (*p* < 0.05, PERMANOVA), indicating that each group harbored compositionally distinct microbial communities (Fig. 5C). MCP group microbiota formed a separate cluster from controls, confirming that hyperprolactinemia shifted the gut ecosystem. Both CPE and PFM groups clustered apart from MCP, each trending back toward the control profile but in different ways. Weighted UniFrac showed less separation between groups (Fig. 5D), suggesting that the dominant taxa remained somewhat consistent but rare taxa changes defined the group differences. Overall, these diversity analyses indicate that CPE and PFM both modulated the dysbiotic microbiota caused by MCP, but possibly with different spectra of action on specific microbial populations.

The bacterial taxa responsible for these differences were subsequently investigated. In MCP-treated mice, several notable shifts were observed relative to healthy controls (Fig. 5E). There was a loss of normally beneficial commensals: genera such as *Faecalibaculum*, *Sellimonas*, and *Olsenella* were significantly decreased by MCP. These bacteria are known anaerobic fermenters, many producing short-chain fatty acids (SCFAs) like acetate and butyrate that support gut health and anti-inflammatory immune responses (Tian et al., 2022). Conversely, MCP-treated mice showed an overgrowth of taxa associated with inflammation and metabolic or mood disorders: *Oscillibacter*, members of the family *Desulfovibrionaceae*, *Alistipes*, *Corynebacterium*, and an unclassified *Lachnospiraceae* (GCA-900066575 group) were all increased. Several of these genera have been linked in literature to negative health outcomes. For instance, *Alistipes, Desulfovibrionaceae* and *Oscillibacter* have repeatedly been found enriched in individuals with depression or depressive-like phenotypes (Liu et al., 2020; Yang et al., 2017). These bacteria may contribute to a pro-inflammatory gut environment; *Oscillibacter* species produce valeric acid (a GABA analog) which might disturb neurotransmitter balance (Kumar et al., 2023), and *Desulfovibrionaceae* are endotoxin-producing sulfate-reducing bacteria known to promote inflammation. The MCP model’s microbial changes thus reflect a dysbiosis that could conceivably exacerbate PMS or mood symptoms, aligning with the notion of a gut-brain-immune connection in PMS (Tian et al., 2020).

**Fig. 6:**
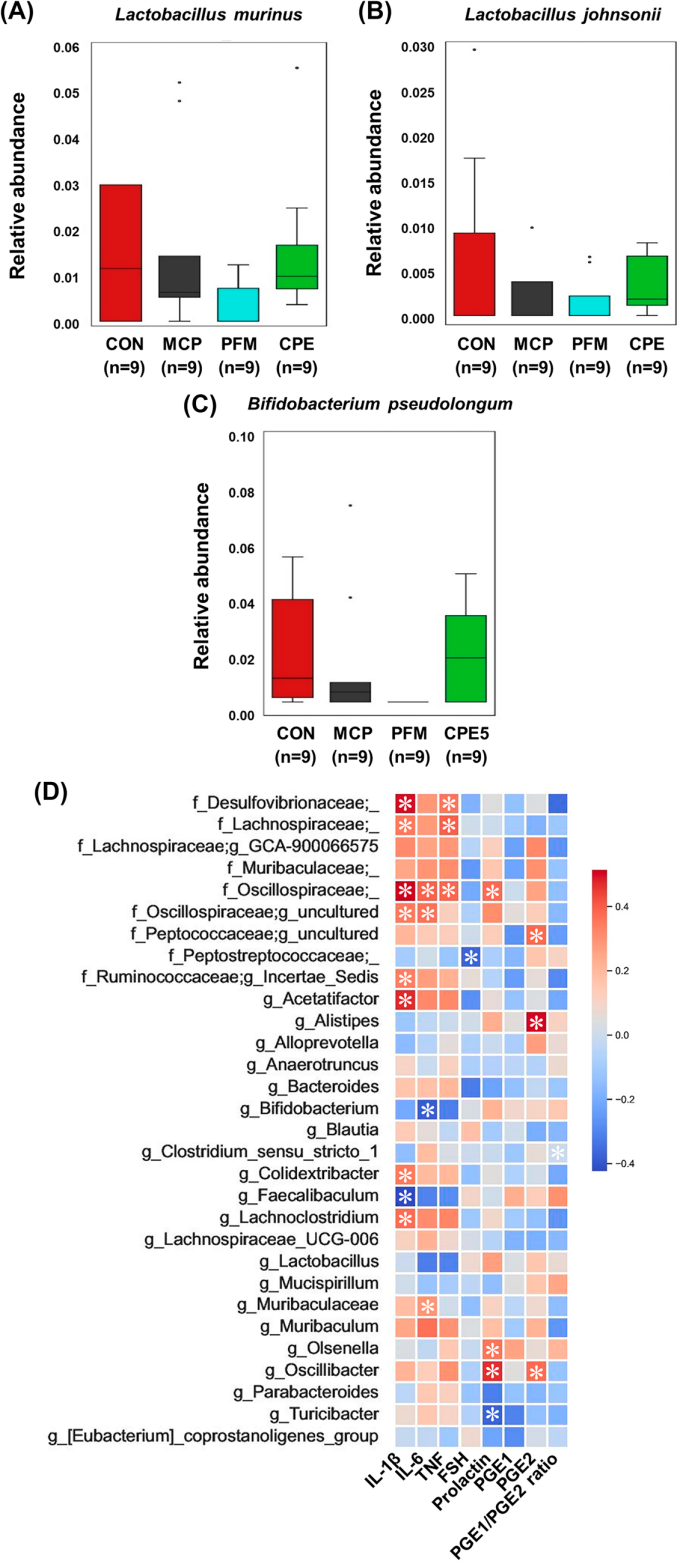
Relative abundance and correlation analysis of intestinal microbiota associated with inflammation and hormone regulation in a PMS mouse model. Species-level abundance of **A** *Lactobacillus murinus*, **B** *Lactobacillus johnsonii*, and **C** *Bifidobacterium pseudolongum* across experimental groups. **D** Correlation heatmap between microbial taxa and PMS-related markers, including inflammatory cytokines (IL-1β, IL-6, TNF-α), hormones (FSH, prolactin), and prostaglandins (PGE1, PGE2, PGE1/PGE2 ratio). Data are presented as mean ± standard deviation. *CON* control group, *MCP* metoclopramide-induced group, *PFM* MCP-induced group treated with prefemin, *CPE* MCP-induced group treated with *Codonopsis lanceolata* 50% ethanol extract

Treatment with PFM and CPE produced distinct microbial modulation patterns. PFM partially reversed some of the MCP-induced dysbiosis. PFM-treated mice showed increases in certain SCFA-producing genera such as *Parabacteroides* and *Turicibacter*, suggesting a restoration of beneficial fermentation capacity. However, PFM further reduced *Bifidobacterium* and *Faecalibaculum*, which were already decreased by MCP treatment. This could indicate an antimicrobial or growth-inhibitory effect of some compounds in *Vitex* on these bacteria. By contrast, CPE yielded a more favorable rebalancing of the microbiota. CPE treatment significantly increased the relative abundance of classic probiotic and beneficial genera, including *Bifidobacterium* (specifically *B. pseudolongum*), *Lactobacillus* (especially *L. murinus* and *L. johnsonii*), and *Faecalibaculum* (Fig. 6A–C). The enrichment of *Bifidobacterium* and *Lactobacillus* is noteworthy, as these genera are well-known for their anti-inflammatory and gut-barrier-supporting effects (Pi et al., 2024). They produce SCFAs and other metabolites that reinforce intestinal integrity and modulate immune responses, often leading to reduced systemic inflammation. The increase in *Faecalibaculum* further suggests improved colonic fermentation and energy harvesting, which can benefit mucosal health. Concurrently, CPE significantly decreased the abundance of several taxa that were abnormally increased by MCP. *Oscillibacter* and *Muribaculaceae* were brought down toward normal levels, and *Desulfovibrionaceae* were also reduced. These suppressive effects on potentially harmful microbes, combined with promotion of beneficial ones, imply that CPE acts as a prebiotic or microbiota-modulating agent. Components of CPE such as inulin-type polysaccharides or certain saponins could selectively stimulate beneficial commensals. Indeed, *Codonopsis* species are rich in polysaccharides that have been reported to shape gut microbiota and improve metabolic outcomes (Zou et al., 2022). Our findings suggest CPE creates a gut environment skewed toward SCFA producers and probiotics, which may contribute to its overall anti-inflammatory and homeostatic effects. In contrast, the more mixed outcome with PFM helping certain beneficial microbes while hindering others highlights that not all PMS treatments equally benefit the gut microbiota. Broad prebiotic-like enhancements in the microbiome due to CPE intake could give it an edge in providing holistic benefits.

To further explore how these microbiota changes, relate to host physiology, correlation analyses were performed between the relative abundances of key taxa and the host biomarkers (inflammatory cytokines, prostaglandins, and hormones). The results revealed insightful associations (Fig. 6D). *Oscillibacter* abundance was positively correlated with serum prolactin and PGE2 levels in other words, mice with higher *Oscillibacter* tended to have higher prolactin and a more pro-inflammatory prostaglandin profile. A similar positive correlation was found for *Olsenella* with prolactin and PGE2. This suggests these bacteria might contribute to or thrive in a high-prolactin, high-PGE2 environment though causality is not established, it indicates a link between dysbiosis and the endocrine-immune imbalance. On the other hand, classic beneficial genera showed negative correlations with inflammatory markers. The proportions of *Faecalibaculum*, *Lactobacillus*, and *Bifidobacterium* were inversely correlated with IL-1β, IL-6, and TNF-α levels; animals with more of these microbes had lower systemic inflammation. These correlations make biological sense, as many *Lactobacilli* and *Bifidobacteria* are known to secrete anti-inflammatory molecules and to strengthen gut barrier function, thereby reducing endotoxin leakage and inflammatory drive (Khokhlova et al., 2012). Notably, *Faecalibaculum* was inversely linked to TNF-α and IL-1β, underscoring the importance of butyrate-producers in restraining inflammation. Additionally, a higher *Lactobacillus* presence correlated with lower IL-6, aligning with reports that probiotics can lower systemic IL-6 and improve mood symptoms in clinical studies (Arifdjanova et al., 2021). Together, these data suggest that the gut microbiota shifts induced by CPE are not merely incidental, but likely play a contributing role in the extract’s anti-inflammatory and prolactin-regulating effects. By promoting beneficial microbes which in turn correlate with reduced cytokines and balanced prostaglandins, CPE may be engaging the gut–brain–immune axis to alleviate PMS-related outcomes. This intriguing gut connection provides a novel dimension to PMS therapy whereas traditional approaches like PFM or pharmacologic dopamine agonists focus on the endocrine aspect, the ability of CPE intake to concurrently nurture a health-promoting microbiome could confer additional benefits, potentially including improved mood and stress resilience via microbiota-derived neurotransmitters and immune modulators. CPE may therefore represent a promising natural intervention for PMS management with multi-modal physiological effects. However, this study has certain limitations. The active compounds responsible for these effects were not identified, and the precise molecular mechanisms remain to be clarified. In addition, due to the lack of a standardized animal model that fully recapitulates the complexity of human PMS, only a hyperprolactinemia-induced mouse model was employed to mimic key endocrine and inflammatory features. Therefore, future studies should include phytochemical characterization of CPE, mechanistic validation, and human clinical trials with comprehensive behavioral assessments to confirm its efficacy and safety.

## Supplementary Information

Below is the link to the electronic supplementary material.

Supplementary file1 (DOCX 175 kb)
