# Supplementary material for: Therapeutic potential of Codonopsis lanceolata peel extract in premenstrual syndrome: insights into hormonal, immune, and microbial interactions
Source: Food Sci Biotechnol. 2025 Oct 17;34(16):4027–38. doi: 10.1007/s10068-025-02003-w (PMC12589743; doi:10.1007/s10068-025-02003-w)
Supplement: Supplementary file 1 — Supplementary file1 (DOCX 175 kb) [file 10068_2025_2003_MOESM1_ESM.docx]

**Supplementary Materials:**

**Cell Viability (MTT) Assay**

Raw 264.7 macrophages were maintained in DMEM containing 10% FBS and 1% penicillin–streptomycin at 37℃ in a 5% CO_2_ atmosphere. GH3 pituitary cells were maintained in F-12K medium supplemented with 15% horse serum, 2.5% FBS, and 1% penicillin–streptomycin under the same conditions. Cells were treated with *C. lanceolata* peel extract (CPE) dissolved in PBS at final concentrations of 0, 50, 100, 250, 500, or 1000 µg/mL for 24 h. Cell viability of CPE was determined by using 3-(4,5-dimethylthiazol-2-yl)-2,5-diphenyltetrazolium bromide (MTT) assay. The absorbance was measured at 590 nm on a microreader (BioTek, Winooski, VT, USA). Viability was expressed relative to the PBS control. No cytotoxicity was observed at 100 µg/mL in either cell line.

**Figures**


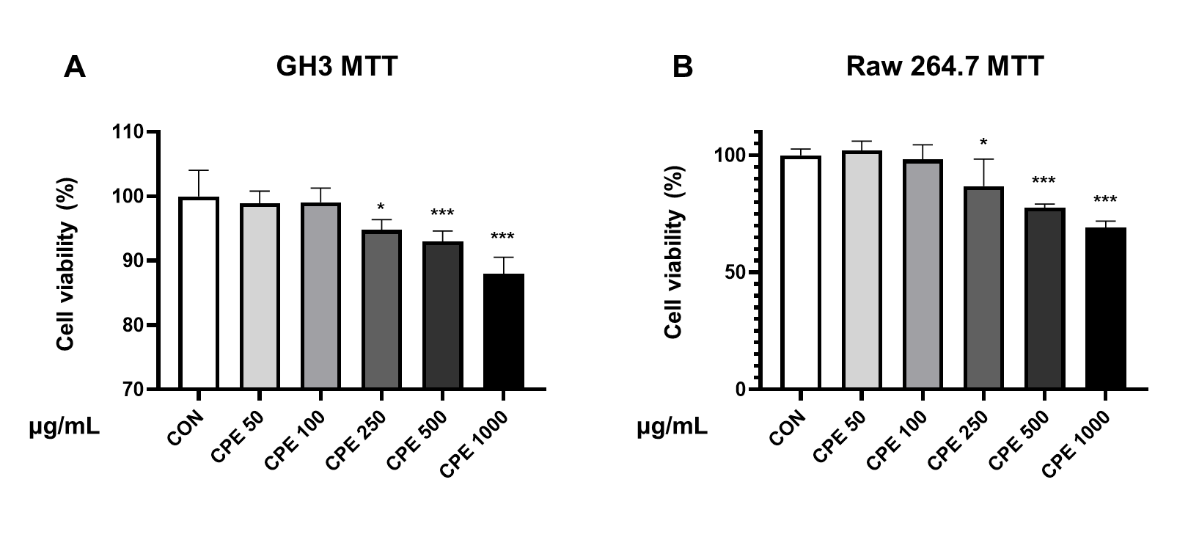


**Figure S1**: Cell viability assessed by MTT assay in (A) GH3 and (B) Raw 264.7 cells. Data are expressed as mean ± SD. * *p* < 0.05, *** *p* < 0.001.
